# Multi-omics reveals functional recovery of the gut microbiome in rescued Sunda pangolins (*Manis javanica*)

**DOI:** 10.1016/j.isci.2026.116754

**Published:** 2026-07-13

**Authors:** Zhidong Zhang, Yan Shu, Xinyu Liu, Bowen Xu, Jiao Chen, Zhenquan Zhang, Kai Wang, Yan Hua

**Affiliations:** 1 Guangdong Provincial Key Laboratory of Silviculture, Protection and Utilization, Guangdong Academy of Forestry, Guangzhou 510520, China; 2 College of Forestry and Landscape Architecture, South China Agricultural University, Guangzhou 510642, China

**Keywords:** Sunda pangolin, rehabilitation, gut microbiome, metagenomics, metabolomics

## Abstract

The Sunda pangolin (*Manis javanica*), a critically endangered myrmecophage, often develops severe gastrointestinal disturbance after trafficking, creating major challenges for post-rescue rehabilitation. We integrated 16S rRNA gene sequencing, shotgun metagenomics, untargeted metabolomics, and gas chromatography-mass spectrometry (GC-MS) quantification of short-chain fatty acids to investigate gut ecosystem recovery in rescued pangolins across the first abnormal fecal stage, 1 week post-rescue, and 1 month post-rescue. Fecal consistency improved during rehabilitation, accompanied by a shift from facultative taxa enriched in *Streptococcus* and *Lactobacillus* to a more anaerobic community containing *Clostridium*, *Romboutsia*, *Bacteroides*, and related taxa. Metagenomic and metabolomic profiles indicated recovery of functions associated with chitin degradation, short-chain fatty acid production, amino acid metabolism, and cofactor biosynthesis. Increased fecal butyrate and multi-omics associations supported recovery of microbial metabolic function. These findings provide insight into the microbial and metabolic dynamics of gut ecosystem recovery in rescued pangolins and may help assess rehabilitation progress in this critically endangered species.

## Highlights

• Multi-omics tracked gut recovery in rescued Sunda pangolins
• Rehabilitation shifted the gut microbiome toward obligate anaerobic taxa
• Microbial genes and SCFAs indicated restored fermentative capacity
• Integrated profiles identified candidate markers of pangolin gut recovery

## Graphical abstract

**Figure:**
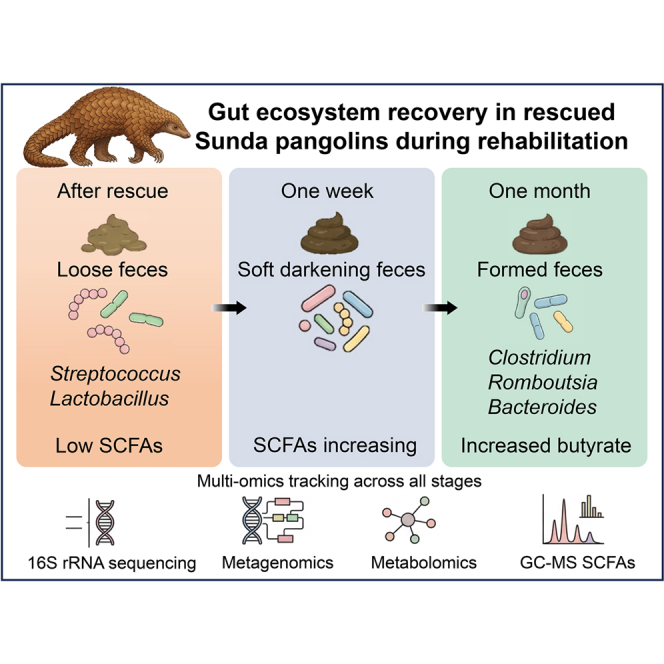

## Teaser

Molecular biology; Microbiology

## Introduction

The illegal wildlife trade is a major driver of global biodiversity loss, pushing numerous species, including the Sunda pangolin (*Manis javanica*), toward extinction. As the world’s most heavily trafficked mammal, the Sunda pangolin is currently classified as critically endangered.^1^ Despite intensified interception and rescue efforts, post-confiscation survival remains alarmingly low, with mortality rates consistently exceeding 67% within the first 10 days following rescue.^2^ This early mortality is frequently associated with severe gastrointestinal distress and systemic organ failure, both of which arise from extreme physical and psychological trauma experienced during trafficking.^2^^,^^3^ Previous reports have suggested that trafficked pangolins are often subjected to non-natural feeding practices, including forced administration of artificial substrates, in attempts to keep them alive or increase body weight before sale, with potentially severe consequences for gastrointestinal function.^2^ Although such trauma disrupts gastrointestinal homeostasis, the biological processes underlying intestinal recovery following acute disturbance remain poorly understood.

Gut microbiota play a fundamental role in host fitness, particularly in dietary specialists with highly constrained nutritional niches.^4^ As obligate myrmecophages, pangolins have evolved a specialized gut microbiome that converges with that of other myrmecophagous mammals, notably harboring taxa adapted to degrade recalcitrant chitin.^5^^,^^6^ Under natural conditions, this co-evolved microbial community is essential for energy acquisition, nutrient assimilation, and intestinal homeostasis. However, such specialization also renders the pangolin gut ecosystem highly sensitive to external perturbations. Recent ecological studies have demonstrated that diet is a primary determinant of microbial structure and function, regulating key enzymatic pathways involved in polysaccharide and protein degradation.^7^ Accordingly, exposure to the abnormal carbohydrate-rich gruels reported in pangolin trafficking would represent a severe and non-physiological stressor to the gastrointestinal system and could contribute to mucosal injury, gut ecosystem destabilization, and impaired intestinal barrier integrity.

To date, research on pangolin gut microbiota has focused primarily on cross-sectional comparisons between wild and captive or rescued individuals, revealing marked differences in microbial composition and predicted functional potential.^8^^,^^9^ While these studies have provided essential baseline insights into captivity-associated microbial alterations, they largely capture static endpoints rather than dynamic recovery processes. Rehabilitation, however, is inherently a temporal process involving progressive physiological and microbial reorganization. Longitudinal studies that track gut ecosystem responses to acute trauma and subsequent dietary transition remain scarce. Moreover, reliance on 16S rRNA gene sequencing alone limits inference regarding functional recovery, as shifts in taxonomic composition do not necessarily reflect restoration of metabolic capacity or microbial-host interactions. This requires integrative approaches to examine changes in microbial structure, functional pathways, and metabolic outputs.^4^

In this study, we applied a multi-omics framework integrating 16S rRNA gene sequencing, shotgun metagenomics, and untargeted metabolomics to investigate gut ecosystem dynamics in rescued Sunda pangolins. By following individuals from the first defecation event after rescue admission through 1-week and 1-month post-rescue stages, we aimed to characterize temporal changes in microbial community structure, functional potential, and metabolic profiles during rehabilitation. This integrative approach provides a comprehensive framework for evaluating intestinal recovery and offers mechanistic insight into gut ecosystem restoration in this critically endangered species.

## Results

### Fecal scoring and morphological assessment

**Figure 1:**
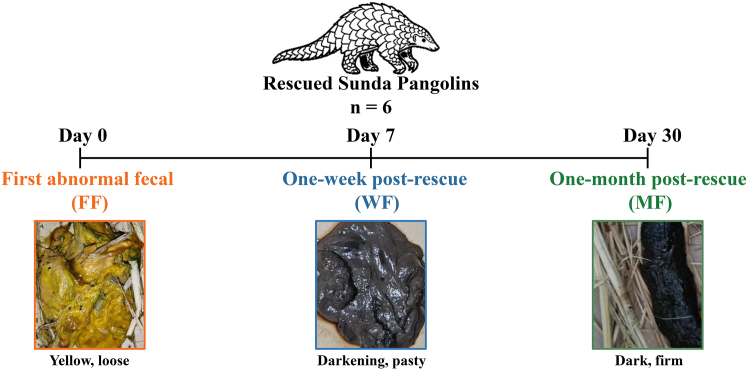
Study timeline and changes in fecal phenotype in rescued Sunda pangolins Fecal samples were collected from *n* = 6 pangolins at day 0 (first abnormal fecal stage, FF), day 7 (1 week post-rescue, WF), and day 30 (1 month post-rescue, MF). Representative images show the transition in fecal characteristics over the 30-day recovery period. The border colors correspond to the respective sampling stages: orange (FF), blue (WF), and green (MF).

Fecal consistency scores (FCSs) were used to quantify changes in gut condition (Table 1). The FF stage showed the highest FCS (4.22 ± 0.44), with yellow, loose, pasty feces lacking defined structure and containing visible undigested material. After 1 week of rehabilitation (WF), fecal consistency improved slightly (3.85 ± 0.55); feces darkened to brown/black but remained soft and pasty. By 1 month post-rescue (MF), FCS decreased markedly to 1.75 ± 0.45, with well-formed, dark, solid stools consistent with grade 2 fecal hardness (Figure 1).

**Table 1:** Fecal consistency scores across recovery stages

Stage	Mean fecal score ± SD
FF	4.22 ± 0.44
WF	3.85 ± 0.55
MF	1.75 ± 0.45

FF, first abnormal fecal stage; WF, 1 week post-rescue; MF, 1 month post-rescue.

### Shifts in gut microbiota diversity and composition

High-throughput sequencing of the 16S rRNA gene yielded sufficient high-quality reads for downstream analysis across all samples (Table S2). Rarefaction curves based on the observed ASVs reached a plateau, indicating sufficient sequencing depth to capture most microbial diversity in the pangolin gut ecosystem (Figure S1A). The Venn diagram illustrates unique and shared taxa among stages (Figure S1B).

**Figure 2:**
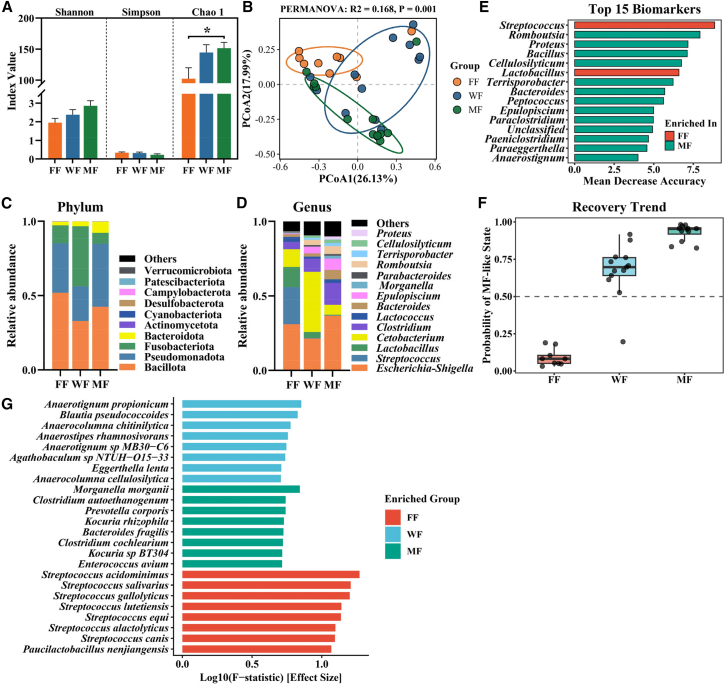
Shifts in gut microbiota diversity and composition in rescued Sunda pangolins (A) Alpha diversity indices (Chao1, Shannon, and Simpson) across recovery stages, analyzed using linear mixed-effects models (LMMs) to account for repeated measurements within individuals (∗adjusted *p* < 0.05). (B) Principal coordinate analysis (PCoA) based on Bray-Curtis dissimilarities showing stage-associated shifts in community structure; group differences were tested by PERMANOVA with permutations constrained within individuals (strata; 999 permutations). (C and D) Taxonomic composition of dominant taxa at the phylum and genus levels. (E) Top 15 discriminatory genera identified by a random forest classifier using leave-one-subject-out cross-validation (LOSO-CV); genera are ranked by mean decrease accuracy, and bar colors indicate enrichment in FF (red) or MF (blue). (F) Microbial Recovery Index (MRI), calculated as the LOSO-CV-predicted probability of a sample resembling the MF-like state; the dashed line indicates the classification threshold (probability = 0.5). (G) Differential abundance of bacterial species among recovery stages identified using LMMs, with features ranked by log-transformed F-statistics. FF, first abnormal fecal stage; WF, 1 week post-rescue; MF, 1 month post-rescue.

Alpha diversity analysis showed that the Chao1 richness index was significantly lower in the FF stage than in the MF stage (*p* < 0.05; Figure 2A), whereas Shannon and Simpson indices did not differ significantly across stages. Beta diversity based on Bray-Curtis distances revealed stage-dependent separation in community structure (PERMANOVA, R^2^ = 0.168, *p* = 0.001; Figure 2B). The first two axes explained 27.01% and 18.6% of the total variance, respectively. Samples from the FF stage clustered distinctly. WF and MF samples partially overlapped, indicating a progressive transition. At the phylum level, Bacillota, Pseudomonadota, Fusobacteriota, and Bacteroidota dominated across all stages (Figure 2C). At the genus level, *Streptococcus*, *Lactobacillus*, and *Escherichia-Shigella* were most abundant during FF, whereas *Cetobacterium* and *Bacteroides* increased during WF. In MF, higher relative abundances of *Clostridium* and *Romboutsia* were observed (Figure 2D). A random forest classifier identified the top 15 genera discriminating among stages (Figure 2E). *Streptococcus* and *Lactobacillus* contributed most to FF classification, while *Romboutsia*, *Bacillus*, *Proteus*, and *Cellulosilyticum* were the strongest predictors associated with MF. Based on the model probabilities, a microbial recovery index indicated low similarity to the MF state during FF (mean probability <0.25), intermediate values in WF (∼0.7), and high resemblance in MF (mean probability >0.9) (Figure 2F).

Species-level analysis of the metagenomic data identified distinct bacterial taxa enriched at each stage (Figure 2G). The FF stage was dominated by various *Streptococcus* species, such as *Streptococcus acidominimus*, *Streptococcus salivarius*, and *Streptococcus gallolyticus*, alongside *Paucilactobacillus nenjiangensis*. During the intermediate WF stage, the community shifted toward obligate anaerobes, with notable increases in *Anaerocolumna* species including *Anaerocolumna chitinilytica* and *Anaerocolumna cellulosilytica*, as well as *Anaerotignum* and *Blautia pseudococcoides*. In the MF recovery stage, the microbiome was characterized by the enrichment of *Bacteroides fragilis*, *Prevotella corporis*, and *Clostridium* species like *Clostridium autoethanogenum* and *Clostridium cochlearium*.

### Functional metagenomic shifts and CAZyme profile remodeling

Shotgun metagenomic sequencing generated a robust dataset for functional annotation. Detailed sequencing statistics and quality control metrics are summarized in Table S3. Following the removal of low-quality reads and host contamination, the resulting high-quality non-host reads were utilized for functional profiling.

**Figure 3:**
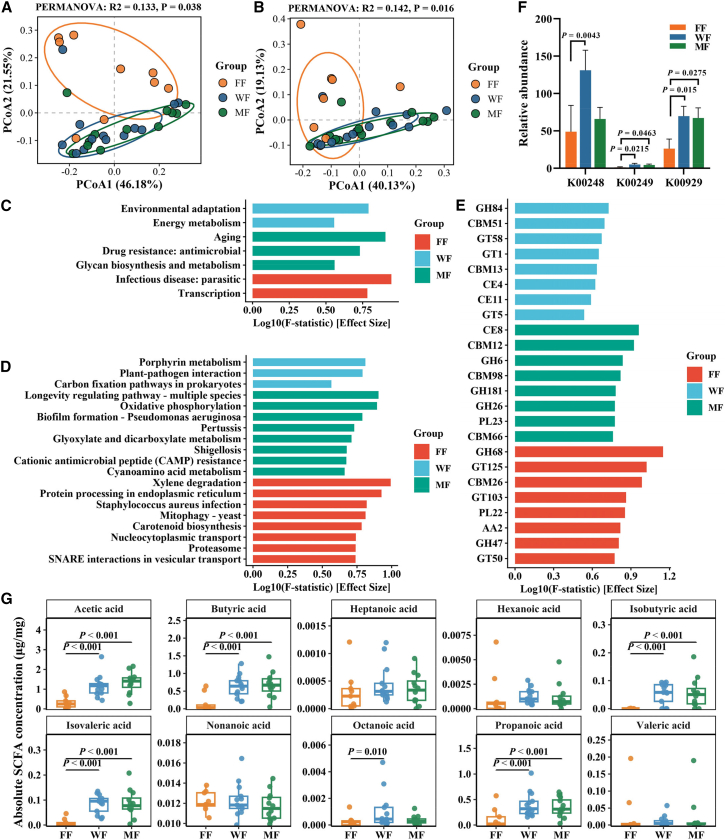
Shifts in gut microbiome functional potential and carbohydrate-active enzyme profile (A and B) PCoA based on Bray-Curtis dissimilarities of KEGG ortholog (KO) profiles and CAZyme profiles, respectively. Group differences were assessed using PERMANOVA with permutations constrained within individuals. (C and D) Differentially abundant KEGG pathways identified using LMMs. The top panel shows enriched KEGG level 2 functional categories, and the bottom panel shows enriched KEGG level 3 pathways across recovery stages. Effect sizes are represented by the log-transformed F-statistics of the group effect. (E) Differential abundance of CAZyme families among recovery stages identified using LMMs, with features ranked by log-transformed F-statistics. (F) Relative abundance of genes involved in butyrate synthesis, including K00248 (butyryl-CoA dehydrogenase), K00249 (acyl-CoA dehydrogenase), and K00929 (butyrate kinase). Group differences were assessed using LMMs. (G) Concentrations of short-chain fatty acids (SCFAs) across recovery stages. Data are presented as mean ± SEM. FF, first abnormal fecal stage; WF, 1 week post-rescue; MF, 1 month post-rescue.

Functional ordination based on KEGG orthologs (KOs) revealed significant separation among stages (PERMANOVA, R^2^ = 0.133, *p* = 0.038; Figure 3A), with FF differing most strongly from WF and MF. CAZyme profiles also showed separation by stage (PERMANOVA, R^2^ = 0.142, *p* = 0.016; Figure 3B), with FF clustering distinctly along the primary axis. Differential analysis based on F-statistics identified stage-specific functions. At KEGG level 2, the FF group was enriched in infectious disease: parasitic and transcription. The WF group was associated with environmental adaptation and energy metabolism. The MF group showed enrichment in aging, drug resistance (antimicrobial), and glycan biosynthesis and metabolism (Figure 3C). At KEGG level 3, the FF microbiome showed higher abundances of pathways including xylene degradation, protein processing in endoplasmic reticulum, *Staphylococcus aureus* infection, and mitophagy (yeast). The WF stage was associated with porphyrin metabolism, plant-pathogen interaction, and carbon fixation pathways in prokaryotes. The MF group was enriched in longevity regulating pathway (multiple species), oxidative phosphorylation, biofilm formation (*Pseudomonas aeruginosa*), and glyoxylate and dicarboxylate metabolism (Figure 3D). Differential CAZyme analysis showed stage-specific differences (Figure 3E). The FF stage was enriched in families such as GH68, GT125, CBM26, and GT103. The WF stage showed higher abundances of GH84, CBM51, GT58, and GT1. The MF group exhibited enrichment in a distinct repertoire, including CE8, CBM12, GH6, CBM98, and GH181. Genes involved in butyrate synthesis increased during recovery (Figure 3F). Compared with the FF stage, the relative abundance of K00248 (butyryl-CoA dehydrogenase) was significantly increased in the WF stage (*p* = 0.0043). K00249 (acyl-CoA dehydrogenase) and K00929 (butyrate kinase) were significantly increased in both the WF stage (*p* = 0.0215 and *p* = 0.0015, respectively) and the MF stage (*p* = 0.0463 and *p* = 0.0275, respectively).

Short-chain fatty acids (SCFAs) were quantified in all 34 fecal samples (Figure 3G). Acetate, propionate, butyrate, isobutyrate, and isovalerate were all significantly increased in the WF and MF stages compared with the FF stage (*p* < 0.001). Nonanoic acid and valeric acid did not differ across stages. Octanoic acid was significantly increased at the WF stage (*p* = 0.010).

### Fecal metabolic profile reflects functional restoration

**Figure 4:**
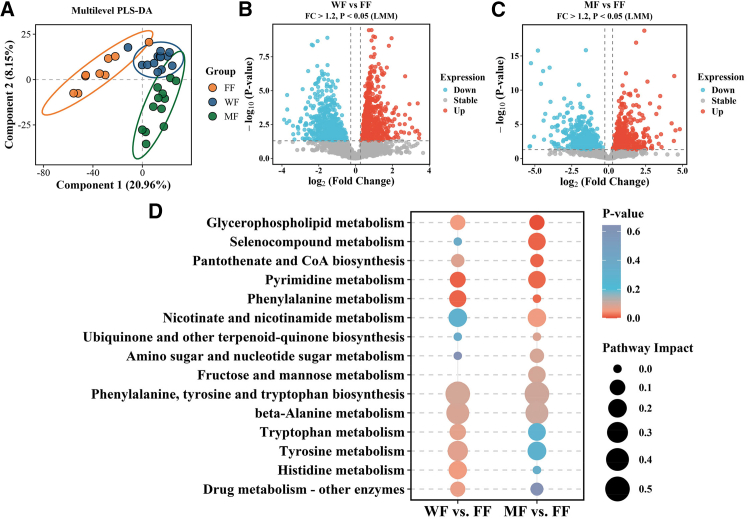
Fecal metabolomic profiles and pathway enrichment analysis across recovery stages (A) Multilevel partial least squares discriminant analysis (multilevel PLS-DA) score plot showing the separation of fecal metabolite profiles among FF, WF, and MF stages, accounting for repeated measurements within individuals. (B and C) Volcano plots illustrating differentially abundant metabolites in the WF vs. FF and MF vs. FF comparisons, respectively. Differential metabolites were identified using linear mixed-effects models (LMMs) and defined by *p* < 0.05 and |log_2_ fold change| > log_2_(1.2). Red and blue points represent significantly increased and decreased metabolites, respectively, while gray points indicate non-significant features. (D) KEGG pathway enrichment and topology analysis of differential metabolites across the recovery stages. The combined bubble plot illustrates the enriched metabolic pathways in the WF vs. FF and MF vs. FF comparisons. Bubble size represents the pathway impact score, and color indicates statistical significance (*p* value). FF, first abnormal fecal stage; WF, 1 week post-rescue; MF, 1 month post-rescue.

Untargeted metabolomics detected a total of 2,948 metabolic features across the different stages. Quality control (QC) assessment demonstrated high instrumental stability and data reproducibility, with QC samples clustering tightly in the PCA score plot and 100% of metabolic features exhibiting a relative standard deviation (RSD) below 30% (Figure S2). To reduce individual variation and improve group separation, we applied Multilevel Partial Least Squares Discriminant Analysis (Multilevel PLS-DA). The score plot showed separation of fecal metabolic profiles (Figure 4A). The FF samples formed a distinct cluster separated from the other groups along component 1 (20.96%), while the WF and MF samples showed a progressive shift, clustering closer together but remaining distinguishable along component 2 (8.15%). To verify the robustness of the observed class separation, a permutation test (*n* = 200) was performed on the PLS-DA model, confirming that the model was not overfitted (Figure S3).

To identify specific metabolites driving these differences, we performed differential analysis using linear mixed-effects models (LMMs) to account for repeated measures [criteria: *p* < 0.05 and |Log_2_FC| > log_2_(1.2)]. This analysis identified substantial metabolite remodeling (Figures 4B and 4C). In the WF vs. FF comparison, 1,118 differential metabolites were identified (37.9% of total features), of which 548 were significantly increased and 570 decreased. Differences were greater in the MF vs. FF comparison, where 1,189 significant differential metabolites were identified, including 549 increased and 640 decreased features.

KEGG pathway enrichment and topology analyses identified stage-specific patterns. Pathway impact and significance values differed across stages (Figure 4D). At the WF stage, the most significantly enriched pathways were pyrimidine metabolism, phenylalanine metabolism, and glycerophospholipid metabolism. Additionally, several amino acid pathways, such as tryptophan metabolism and tyrosine metabolism, exhibited notable pathway impacts during this early phase. By the MF stage, metabolic alterations were characterized by the continuous enrichment of pyrimidine and glycerophospholipid metabolism, together with enrichment of selenocompound metabolism and pantothenate and CoA biosynthesis.

### Integrated multi-omics network analysis of the gut ecosystem

**Figure 5:**
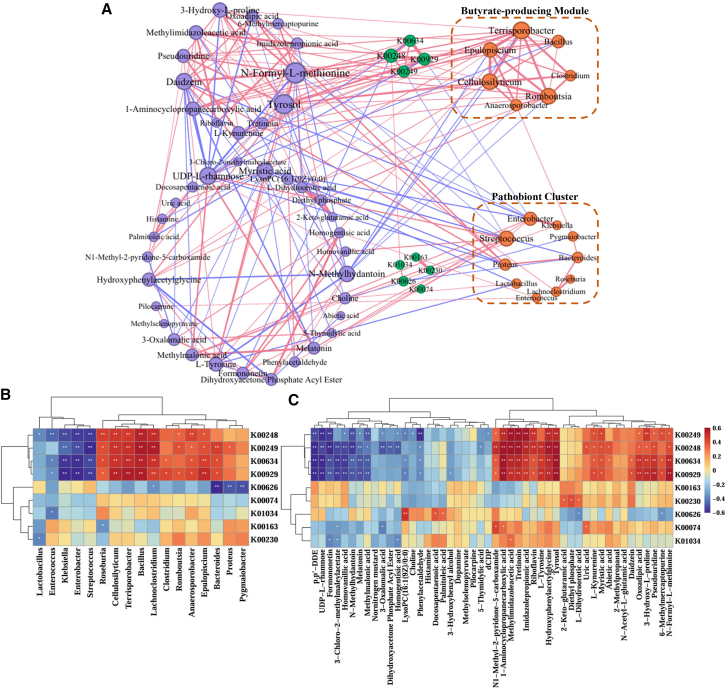
Integrated multi-omics network and association analysis (A) Multi-omics co-occurrence network based on Spearman’s rank correlations (|ρ| ≥ 0.6, false discovery rate [FDR]-adjusted *p* < 0.05). Nodes are colored by category: microbial genera (orange), KEGG orthologs (green), and fecal metabolites (purple). Node size is proportional to the degree of connectivity. Edges indicate significant associations, with red and blue lines representing positive and negative correlations, respectively. Dashed boxes indicate two functional clusters: the butyrate-producing module and the pathobiont module. (B and C) Heatmaps visualizing Spearman correlations between key bacterial genera and KEGG orthologs and differential metabolites and KEGG orthologs. The color gradient represents the correlation coefficient, ranging from negative (blue) to positive (red). Asterisks denote statistical significance (∗adjusted *p* < 0.05, ∗∗adjusted *p* < 0.01).

A co-occurrence network was constructed based on pairwise Spearman correlations among microbial taxa, functional genes (KEGG orthologs), and fecal metabolites across samples (Figure 5A). The resulting network displayed a modular structure with two major clusters. The butyrate-producing module contained *Clostridium*, *Romboutsia*, *Terrisporobacter*, *Cellulosilyticum*, and *Anaerosporobacter*, which were positively correlated with butyrate synthesis genes (K00248, K00249, K00634, and K00929) and metabolites including L-tyrosine, tyrosol, L-kynurenine, N-formyl-L-methionine, and riboflavin. In contrast, the pathobiont module comprised *Enterococcus*, *Klebsiella*, *Enterobacter*, *Streptococcus*, and *Proteus*, which correlated positively with a distinct metabolite set but showed negative associations with butyrate synthesis genes.

Consistent with the network topology, pairwise correlation heatmaps (Figures 5B and 5C) confirmed that *Clostridium*, *Romboutsia*, *Roseburia*, and *Terrisporobacter* were significantly positively correlated with K00248, K00249, K00634, and K00929 (*p* < 0.05), whereas *Lactobacillus*, *Enterococcus*, *Klebsiella*, and *Enterobacter* were negatively correlated. At the metabolite-gene level, butyrate synthesis genes (K00248, K00249, and K00929) were positively associated with N-formyl-L-methionine, L-kynurenine, tyrosol, and riboflavin and negatively associated with UDP-L-rhamnose and formononetin.

## Discussion

### Fecal morphology as an indicator of gastrointestinal recovery

Fecal morphology serves as a primary, non-invasive proxy for assessing gastrointestinal functional restoration during pangolin rehabilitation. The progressive normalization of stool form reflects recovery of intestinal function following earlier gastrointestinal dysfunction. This reflects osmotic imbalance and increased intestinal transit. As FCSs improved across recovery stages, with stools becoming darker and firmer, this suggests prolonged chyme retention and enhanced water reabsorption, consistent with partial restoration of intestinal function.^10^ Associations between fecal consistency and gut microbial structure have also been reported in other mammals, supporting the use of fecal morphology as an integrative indicator of gut health.^11^

Fecal morphology can serve as a simple and low-cost indicator in rescue settings, allowing rapid assessment of gut condition during early rehabilitation. In many cases where molecular tools are not available, standardized fecal scoring can help staff judge whether intestinal function is improving and whether the current diet is appropriate. Fecal phenotype should be considered an initial indicator rather than a direct measure of microbiome or metabolic recovery. It is most useful for identifying animals that require closer observation or supportive care in the early stage.

### Rehabilitation is associated with a shift from facultative to obligate anaerobic gut microbiota

Rehabilitation involved a transition from microbial communities dominated by facultative anaerobes at intake to communities enriched in obligate anaerobic taxa at later stages of recovery. Similar patterns occur in disturbed gut ecosystems, where physiological stress or dietary disruption alters the intestinal environment and favors organisms that tolerate oxygen exposure. Facultative anaerobic bacteria are particularly suited to such conditions because they can exploit metabolic niches created by inflammation or increased epithelial oxygen diffusion.^12^ The dominance of *Streptococcus*, *Lactobacillus*, and *Escherichia-Shigella* observed at intake reflects microbial responses to acute intestinal disturbance following trafficking and dietary mismatch. As rehabilitation progressed, the microbial community shifted toward obligate anaerobic taxa including *Romboutsia*, *Clostridium*, and *Bacteroides*, groups typical of stable fermentative gut ecosystems.^13^ These taxa have also been reported in wild pangolins and are thought to participate in the degradation of insect-derived substrates, a key component of the pangolin diet.^8^^,^^9^ Ecological succession of this type occurs in host-associated microbiomes recovering from disturbance, with communities reorganizing toward host-adapted configurations.^14^ The increasing prevalence of obligate anaerobic fermentative taxa is consistent with a shift toward a microbiome better suited to the pangolin’s insectivorous diet.

### Recovery of microbial functions related to short-chain fatty acid synthesis and chitin degradation

Microbial composition changes coincided with shifts in metabolic functions. Genes involved in butyrate synthesis increased during recovery. Fecal butyrate, together with acetate and propionate, increased significantly in the WF and MF stages, consistent with recovery of microbial fermentation activity. Butyrate is a key end product of microbial fermentation in mammalian intestines and serves as an important energy source for colonocytes while contributing to epithelial barrier maintenance and immune regulation.^15^^,^^16^ Increased abundance of genes associated with acetyl-CoA-dependent butyrate synthesis paralleled the rise of obligate anaerobic taxa. Members of Bacillota, including *Clostridium*-related lineages, are major contributors to butyrate production in mammalian gut ecosystems.^13^

In addition to SCFA metabolism, metagenomic data indicated recovery of microbial functions related to chitin degradation. Chitin is a major structural polysaccharide in insect exoskeletons and represents a central component of the pangolin diet. Because pangolins possess limited endogenous chitinase capacity, microbial symbionts are thought to play an important role in chitin hydrolysis and downstream nutrient assimilation.^17^ The recovering microbiome exhibited increased abundance of carbohydrate-active enzyme families associated with chitin processing, including chitin-binding modules such as CBM12. Comparable microbial adaptations have been documented in the gut microbiota of other myrmecophagous mammals that rely on insect-based diets.^18^ Rehabilitation reflects gradual reconstruction of microbial metabolic functions related to fermentation and chitin degradation. This recovery may improve the capacity of the gut microbiome to utilize insect-derived substrates and support host energy metabolism.

### Metabolomic signatures indicate progressive normalization of intestinal metabolism

Metabolomic profiling revealed substantial changes in intestinal metabolic activity during the rehabilitation period. Early stages of recovery showed enrichment of pathways related to lipid remodeling and amino acid metabolism, including glycerophospholipid and phenylalanine metabolism. Similar metabolic responses have been reported during recovery from starvation or severe physiological stress, when cellular repair processes and membrane turnover increase as tissues resume metabolic activity.^19^ These features reflect initial physiological adjustment of the intestinal system following the disruption associated with trafficking and dietary mismatch. As rehabilitation progressed, metabolic profiles shifted toward pathways involved in cofactor production and broader cellular metabolism. Enrichment of pantothenate and coenzyme A biosynthesis indicates increasing availability of metabolic cofactors required for fatty acid oxidation and energy metabolism. Similar metabolic adjustments occur in animals adapting to protein-rich or insect-based diets, where efficient utilization of protein and chitin requires coordinated microbial and host metabolic activity.^20^^,^^21^ Changes in metabolites linked to microbial amino acid metabolism were also detected during later recovery stages, consistent with the restructuring of the gut microbiome observed during rehabilitation.

These metabolic shifts indicate a transition from a stress-associated state to a more stable intestinal metabolic environment. The metabolite profiles indicate interactions between host metabolism and microbial fermentation in the gut. These metabolic changes characterize the progression of intestinal recovery in rescued pangolins.

### Multi-omics integration highlights coordinated changes in microbial structure and function

Integrated analysis of microbial, metagenomic, and metabolomic data showed that changes in microbial community composition were accompanied by shifts in functional genes and metabolic profiles. Microbial taxa that increased during later recovery stages were positively associated with genes involved in butyrate synthesis, linking microbial restructuring to fermentative metabolic pathways. Associations between microbial taxa and metabolites reflect shifts in metabolic activity within the gut ecosystem. Several metabolites related to amino acid metabolism and vitamin synthesis were positively correlated with obligate anaerobic taxa and butyrate-related genes. Similar relationships between microbial composition and metabolic profiles have been described in mammalian gut systems, where microbial fermentation products influence the intestinal metabolic environment.^22^^,^^23^

Coordinated changes across microbial composition, functional genes, and metabolites indicate that recovery of the pangolin gut ecosystem involves reorganization across multiple biological levels. The recovering microbiome is not only taxonomically different from the initial dysbiotic state but also functionally and metabolically distinct.

### Limitations of the study

This study has several limitations. First, the cohort size was small due to the constraints of working with a critically endangered species during rescue. The repeated-measures design and mixed-effects models improved robustness, but the results require validation in larger and independent cohorts. Second, this was an observational study. The taxa, pathways, and metabolites identified here should be considered as recovery-associated patterns rather than direct drivers of recovery. Third, metagenomic data describe functional potential but not actual activity. Further functional assays are needed to evaluate microbial activity.

This study identifies candidate microbial and metabolic indicators that may help monitor rehabilitation progress in rescued pangolins. The Microbial Recovery Index (MRI), fecal scoring, and selected microbial and metabolic features may serve as useful indicators of recovery, although they are not yet validated for clinical use. Future work should test whether microbiome or metabolome profiles at intake can predict short-term survival and whether these markers can help guide dietary transitions and improve rescue decisions. Our results show that recovery involves more than clinical stabilization and includes rebuilding a gut ecosystem suited to the host’s specialized diet.

## Resource availability

### Lead contact

Requests for further information and resources should be directed to and will be fulfilled by the lead contact, Yan Hua (wildlife530@hotmail.com).

### Materials availability

This study did not generate new unique reagents.

### Data and code availability

The 16S rRNA gene sequencing and metagenomic data have been deposited in the NCBI Sequence Read Archive (SRA) and are available under NCBI BioProject: PRJNA1392139. The metabolomics data are available in the CNGB Sequence Archive (CNSA) under CNSA: CNP0008713. This paper does not report original code. Any additional information required to reanalyze the data reported in this paper is available from the lead contact upon reasonable request.

## Acknowledgments

We thank the Guangdong Provincial Wildlife Monitoring and Rescue Center for assistance with pangolin rehabilitation and sample collection. This work was funded by 10.13039/501100021171Guangdong Basic and Applied Basic Research Foundation (2024A1515012617).

## Author contributions

Conceptualization, K.W. and Y.H.; methodology, K.W. and Y.H.; investigation, Zhidong Zhang, Y.S., X.L., B.X., J.C., and Zhenquan Zhang; formal analysis, Zhidong Zhang; data curation, Zhidong Zhang; visualization, Zhidong Zhang; writing – original draft, Zhidong Zhang; writing – review and editing, K.W. and Y.H.; resources, Y.H.; funding acquisition, Y.H.; supervision, K.W. and Y.H.

## Declaration of interests

The authors declare no competing interests.

## STAR★Methods

### Key resources table

**Table:** 

REAGENT or RESOURCE	SOURCE	IDENTIFIER
**Biological samples**		

Fecal samples from rescued Sunda pangolins	This paper	N/A

**Chemicals, peptides, and recombinant proteins**		

Formic acid	DIMKA	Cat# 50144-50 mL
Ammonium formate	Honeywell Fluka	Cat# 17843-250G
Methanol	Thermo Fisher Scientific	Cat# A454-4
Acetonitrile	Thermo Fisher Scientific	Cat# A998-4

**Critical commercial assays**		

MagPure Stool DNA KF Kit B	Magen, China	Cat# MD5115
Agencourt AMPure XP magnetic beads	Beckman Coulter	Cat# A63880

**Deposited data**		

16S rRNA gene sequencing data	This paper	NCBI BioProject: PRJNA1392139
Shotgun metagenomic sequencing data	This paper	NCBI BioProject: PRJNA1392139
Untargeted metabolomics data	This paper	CNSA: CNP0008713
*Manis javanica* reference genome	NCBI	GCF_040802235.1
SILVA reference database	Quast et al.^24^	https://www.arb-silva.de
KEGG database	Kanehisa Laboratories	https://www.kegg.jp
eggNOG database	eggNOG	http://eggnog.embl.de
CAZy database	CAZy	http://www.cazy.org
CARD database	CARD	https://card.mcmaster.ca
BGI Metabolome Database	BGI	N/A
mzCloud database	Thermo Fisher Scientific	https://www.mzcloud.org
ChemSpider database	Royal Society of Chemistry	https://www.chemspider.com
Unified Human Gastrointestinal Genome collection	Almeida et al.^25^	N/A

**Experimental models: Organisms/strains**		

Sunda pangolin (*Manis javanica*)	This paper	NCBI: txid9974

**Oligonucleotides**		

16S rRNA gene V3–V4 fusion primers	This paper	341F: 5′-CCTACGGGNGGCWGCAG-3′; 806R: 5′-GGACTACHVGGGTWTCTAAT-3′

**Software and algorithms**		

QIIME 2	Bolyen et al.^26^	https://qiime2.org
DADA2	Callahan et al.^27^	N/A
FLASH	Magoč and Salzberg^28^	N/A
SOAPnuke	Chen et al.^29^	N/A
Bowtie2	Langmead and Salzberg^30^	http://bowtie-bio.sourceforge.net/bowtie2
MEGAHIT	Li et al.^31^	https://github.com/voutcn/megahit
MetaGeneMark	Zhu et al.^32^	N/A
CD-HIT	Fu et al.^33^	https://github.com/weizhongli/cdhit
Salmon	Patro et al.^34^	https://salmon.readthedocs.io
DIAMOND	Buchfink et al.^35^	https://github.com/bbuchfink/diamond
Kraken2	Wood et al.^36^	https://github.com/DerrickWood/kraken2
Bracken	Lu et al.^37^	https://github.com/jenniferlu717/Bracken
Compound Discoverer v3.3	Thermo Fisher Scientific	https://mycompounddiscoverer.com/
metaX	Wen et al.^38^	N/A
R v4.2.3	R Project	https://www.r-project.org
mixOmics package	mixOmics	http://mixomics.org
psych package	CRAN	https://cran.r-project.org/package=psych
Gephi	Gephi Consortium	https://gephi.org

**Other**		

Illumina NovaSeq 6000 platform	Illumina	N/A
DNBSEQ platform	MGI Tech, China	N/A
Covaris acoustic shearing system	Covaris	N/A
NanoDrop 2000 spectrophotometer	Thermo Fisher Scientific	N/A
Agilent 2100 Bioanalyzer	Agilent Technologies	Cat# G2939AA
UHPLC system	Thermo Fisher Scientific	N/A
Q Exactive HF mass spectrometer	Thermo Fisher Scientific	N/A
ACQUITY UPLC BEH C18 column, 1.7 μm, 2.1 × 100 mm	Waters	Cat# 186002352
Agilent 7890B gas chromatograph	Agilent Technologies	N/A
Agilent 5977B mass spectrometer	Agilent Technologies	N/A
HP-FFAP capillary column, 30 m × 250 μm × 0.25 μm	Agilent Technologies	Cat# 19091F-433E

### Experimental model and study participant details

#### Ethics committee and approval

This study complies with the Regulations on the Administration of Laboratory Animals established by the National Science and Technology Commission of the People’s Republic of China, as well as the guidance issued by the Ministry of Science and Technology on the treatment of laboratory animals and other relevant regulations. All methods in this study were carried out with the approval of the Experimental Animal Management and Ethics Committee of the Guangdong Academy of Forestry (No. 202400802).

#### Animals

This study included six rescued adult Sunda pangolins (*Manis javanica*), comprising four males and two females. The animals were confiscated by local wildlife law enforcement authorities from illegal wildlife trade cases in Guangdong Province, China, and transferred to the Guangdong Provincial Wildlife Monitoring and Rescue Center in Guangzhou for rehabilitation. Individual sex, developmental stage, and sampling details are provided in Table S1. Detailed pre-rescue information, including diet, trafficking duration, stress exposure, and medical history, was unavailable because the animals originated from law-enforcement confiscations. During rehabilitation, animals were maintained under standardized captive conditions and provided with a formulated artificial diet and *ad libitum* access to water. Given the small cohort size, sex-associated effects on gut microbiome recovery were not formally assessed.

### Method details

#### Sample collection and DNA extraction

Six Sunda pangolins (*Manis javanica*) were confiscated by local wildlife law enforcement authorities from illegal wildlife trade cases in Guangdong Province, China, and subsequently transferred to the Guangdong Provincial Wildlife Monitoring and Rescue Center (Guangzhou, China) for rehabilitation. Upon arrival, animals were maintained under standardized captive conditions and provided with a formulated artificial diet and *ad libitum* access to water. During the first night after admission, all six individuals produced yellow, loose, and pasty feces containing undigested fibrous residues, suggesting gastrointestinal disturbance at intake.

A stage-based longitudinal sampling scheme was used to monitor stool normalization together with gut microbiome and metabolome recovery during rehabilitation. The FF stage was operationally defined as the first abnormal fecal stage observed after rescue admission, corresponding to samples collected from the first defecation event following intake at the rescue center. Such fecal characteristics differ markedly from those typically produced under the natural insect-based diet of pangolins and were therefore considered indicative of severe dietary disturbance prior to rescue. Although the exact pre-confiscation feeding history could not be independently verified, this stage represents the earliest observable gastrointestinal condition at admission.

Fecal samples were collected at three recovery stages to characterize longitudinal gut microbiome dynamics: (i) the first abnormal fecal stage (FF), corresponding to samples collected from the first defecation event after admission; (ii) the one-week post-rescue stage (WF), representing a transitional phase with progressively darkening feces; and (iii) the one-month post-rescue stage (MF), representing a stabilized recovery phase with dark, firm, and well-formed feces (Figure 1).

A total of 34 fecal samples were collected. Because defecation frequency varied among individuals during rehabilitation, additional fecal samples were occasionally collected from some individuals within the same stage (Table S1). These repeated collections captured short-term variability in gut condition within each stage. Fresh feces were obtained within 2 h of defecation. To minimize environmental contamination, the outer layer of each fecal sample was removed using sterile swabs, and material from the inner core was collected. Samples were aliquoted into sterile 2-mL cryotubes and immediately snap-frozen in liquid nitrogen before storage at −80°C.

Total genomic DNA was extracted from approximately 200 mg of fecal material using the MagPure Stool DNA KF Kit B (Magen, China) following the manufacturer’s protocol. DNA concentration and purity were assessed using a NanoDrop 2000 spectrophotometer (Thermo Fisher Scientific, USA), and DNA integrity was verified by 1% agarose gel electrophoresis. Extracted DNA was stored at −20°C until further analysis.

#### Assessment of fecal consistency

Fecal consistency was evaluated at each recovery stage using a standardized five-point fecal scoring system developed for captive myrmecophagous mammals.^39^ Scores ranged from Grade 1 (hard, dry pellets) to Grade 5 (watery diarrhea), with Grade 2 (firm, well-formed, and moist feces) considered optimal for healthy pangolins. All assessments were conducted by a single trained observer to minimize subjective bias.

#### 16S rRNA gene amplicon sequencing and analysis

16S rRNA gene sequencing was performed on 34 fecal samples (Table S2). The V3-V4 hypervariable regions of the bacterial 16S rRNA gene were amplified using primers 341F and 806R with 30 ng of template DNA. Amplicons were purified using Agencourt AMPure XP magnetic beads (Beckman Coulter, USA), and library quality and fragment size were assessed using an Agilent 2100 Bioanalyzer. Sequencing was performed on an Illumina NovaSeq 6000 platform to generate 2 × 250 bp paired-end reads. Raw reads were quality-filtered using a sliding-window approach (25 bp window, average quality score <20). Reads shorter than 75% of the original length, containing ambiguous bases, or adapter contamination were discarded. Paired-end reads were merged using FLASH.^28^ Amplicon sequence variants (ASVs) were inferred using the DADA2 algorithm implemented in QIIME2.^26^^,^^27^ Taxonomic assignment was conducted against the SILVA reference database.^24^ Alpha diversity indices (Shannon, Simpson, and observed richness) and Bray–Curtis dissimilarities were calculated to assess microbial community structure.

#### Metagenomic sequencing and analysis

Shotgun metagenomic sequencing was performed on 34 fecal samples (Table S3). For metagenomic sequencing, 1 μg of high-quality DNA per sample was fragmented to an average size of 200–400 bp using a Covaris acoustic shearing system. Libraries were constructed following manufacturer instructions and sequenced on the DNBSEQ platform (MGI Tech, China) to generate 150 bp paired-end reads. Raw reads were quality-filtered using SOAPnuke.^29^ Host-derived sequences were removed by mapping reads to the *Manis javanica* reference genome using Bowtie2.^30^ Clean, non-host reads were assembled *de novo* into contigs (≥300 bp) using MEGAHIT.^31^ Open reading frames were predicted using MetaGeneMark,^32^ and a non-redundant gene catalog was constructed using CD-HIT at 95% sequence identity.^33^ Gene abundance was quantified by mapping reads to the gene catalog using Salmon.^34^ Functional annotation was performed by aligning predicted proteins against the KEGG, eggNOG, CAZy, and CARD databases using DIAMOND.^35^ Taxonomic profiling was conducted using Kraken2,^36^ with abundance re-estimation performed using Bracken.^37^ Reference genome support included the Unified Human Gastrointestinal Genome collection.^25^

#### Untargeted metabolomics analysis

Untargeted metabolomics profiling was performed on 34 fecal samples. Fecal samples were homogenized, and metabolites were extracted using a methanol–acetonitrile mixture containing internal standards (d3-leucine, ^13^C_9_-phenylalanine, d5-tryptophan, and ^13^C_3_-progesterone) to ensure analytical stability and data quality. After centrifugation, the supernatants were collected for metabolomic profiling. Untargeted metabolomic analysis was performed using an ultra-high-performance liquid chromatography (UHPLC) system coupled to a Q Exactive HF mass spectrometer (Thermo Fisher Scientific, USA). Chromatographic separation was conducted on a Waters BEH C18 column (1.7 μm, 2.1 × 100 mm) maintained at 45°C. The mobile phase consisted of 0.1% formic acid in water (A) and 0.1% formic acid in methanol (B) for positive ion mode (ESI+), and 10 mM ammonium formate in water (A) and 95% methanol containing 10 mM ammonium formate (B) for negative ion mode (ESI−). The mass spectrometer was operated in data-dependent acquisition mode with a scan range of *m/z* 70–1050. Full MS scans were acquired at a resolution of 120,000, followed by MS/MS scans at a resolution of 30,000.

Raw data were processed using Compound Discoverer 3.3 (Thermo Fisher Scientific). Feature detection and retention time alignment were performed in Compound Discoverer, and metabolite annotation was conducted by searching the BGI Metabolome Database and cross-referencing the results with the mzCloud and ChemSpider databases. Metabolite identification was conducted using a precursor mass tolerance of <5 ppm and a fragment mass tolerance of <10 ppm. Data normalization and signal drift correction were conducted using probabilistic quotient normalization and QC-based robust LOESS signal correction,^40^ and downstream processing was performed using the metaX package.^38^

#### Targeted quantification of short-chain fatty acids (SCFAs)

Short-chain fatty acids (SCFAs) were quantified in all 34 fecal samples using a gas chromatography-mass spectrometry (GC-MS) platform. Fecal samples (approximately 100 mg) were homogenized in 1 mL of ultrapure water, followed by bead-beating and ultrasonic extraction. After centrifugation at 4°C (5000 rpm, 20 min), 0.8 mL of supernatant was transferred to a fresh tube. Sulfuric acid (0.1 mL, 50%) and 0.8 mL of extraction solution (methyl *tert*-butyl ether containing 2-methylvaleric acid as an internal standard, 25 mg/L) were added. Samples were vortexed, shaken, and ultrasonicated in an ice-water bath, followed by incubation and a second centrifugation (4°C, 12,000 rpm, 15 min). The final supernatant was collected for GC-MS analysis.

SCFAs were analyzed using an Agilent 7890B gas chromatograph coupled with a 5977B mass spectrometer (Agilent Technologies, USA) equipped with an HP-FFAP capillary column (30 m × 250 μm × 0.25 μm). Helium was used as the carrier gas at a flow rate of 1.2 mL/min. A 1 μL aliquot was injected in split mode (5:1). The oven temperature program started at 50°C, followed by stepwise increases to 240°C under optimized ramp conditions. The injector, transfer line, ion source, and quadrupole temperatures were set at 220°C, 240°C, 230°C, and 150°C, respectively. The mass spectrometer was operated in electron impact (EI) mode at 70 eV.

Quantification was performed using external calibration curves constructed with authentic standards of individual SCFAs. The concentration of each metabolite was calculated based on peak areas and normalized to sample weight. Quality control samples were analyzed throughout the run to ensure analytical stability and reproducibility.

### Quantification and statistical analysis

Given the unbalanced repeated-measures design, statistical approaches accounting for within-subject dependency were applied throughout the analyses. Statistical significance was defined as *p* < 0.05. All statistical analyses were performed in R (version 4.2.3).

Microbial alpha diversity indices, as well as differential microbial taxa, functional pathways, metabolites, and short-chain fatty acids (SCFAs), were analyzed using linear mixed-effects models (LMMs). In these models, recovery stage was treated as a fixed effect and individual identity as a random effect. Beta diversity was assessed using Bray–Curtis dissimilarity and visualized by principal coordinate analysis (PCoA). Group-level differences in community composition were evaluated using permutational multivariate analysis of variance (PERMANOVA) with 999 permutations, with individual identity specified as the permutation strata. To identify features discriminating recovery stages while accounting for repeated measurements, multilevel partial least squares discriminant analysis (Multilevel PLS-DA) was performed using the mixOmics package. For metabolomic data, fold changes were calculated and log_2_-transformed. Differential metabolites were defined as those with *p* < 0.05 and |Log_2_FC| > log_2_(1.2), and visualized using volcano plots. Random forest models were constructed to identify microbial biomarkers associated with recovery. A leave-one-subject-out cross-validation strategy was applied to avoid data leakage due to repeated measurements. A microbial recovery index (MRI) was calculated following the framework described by Subramanian et al.^41^ Correlations among microbial taxa, functional genes (KEGG orthologs), and fecal metabolites were assessed using Spearman’s rank correlation across samples. Correlation coefficients and Benjamini–Hochberg FDR-adjusted *p* values were computed using the corr.test function in the psych package. Significant correlations were visualized as a heatmap, with significance annotated as ∗ adjusted *p* < 0.05 and ∗∗ adjusted *p* < 0.01. Correlations meeting |ρ| ≥ 0.6 and FDR-adjusted *p* < 0.05 were retained to construct a co-occurrence network. Nodes represented microbial taxa, KEGG orthologs, or metabolites, and edges represented significant positive or negative correlations. The network was visualized in Gephi.

### Additional resources

This study did not generate additional resources.
