# Supplementary material for: Multi-omics reveals functional recovery of the gut microbiome in rescued Sunda pangolins (Manis javanica)
Source: iScience. 2026 Jul 13;29(8):116754. doi: 10.1016/j.isci.2026.116754 (PMC13382273; doi:10.1016/j.isci.2026.116754)
Supplement: Document S1. Figures S1–S3 and Tables S1–S3 [file mmc1.pdf]

iScience, Volume *29*

Supplemental information

Multi-omics reveals functional recovery of the gut microbiome in rescued Sunda pangolins (*Manis javanica*)

Zhidong Zhang, Yan Shu, Xinyu Liu, Bowen Xu, Jiao Chen, Zhenquan Zhang, Kai Wang, and Yan Hua

Supplemental Figures and Legends

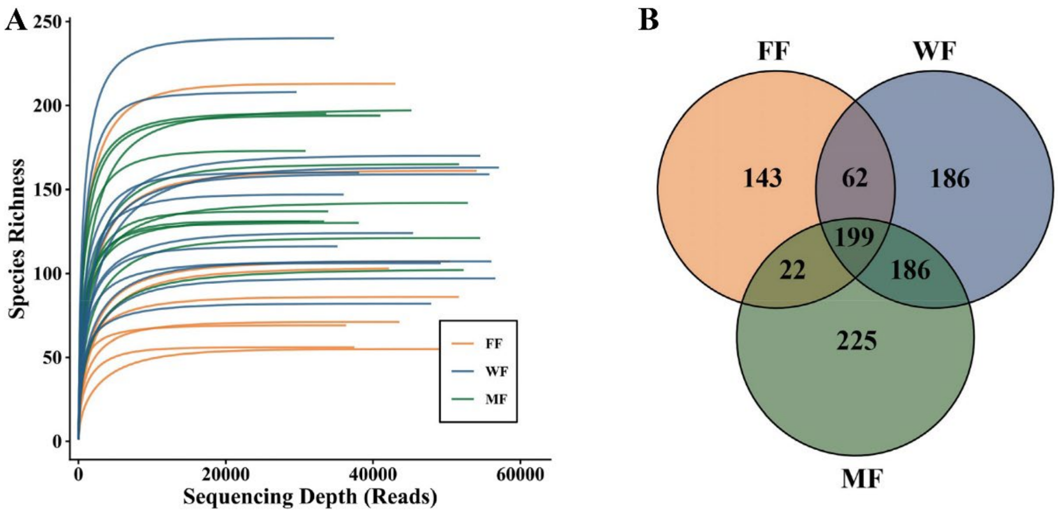

Figure S1. Rarefaction curves and Venn diagram of bacterial ASVs. (A) Rarefaction curves indicate sufficient sequencing depth for all samples. (B) Venn diagram displays the number of common and unique ASVs among the FF, WF, and MF stages. FF, first abnormal fecal stage; WF, one-week post-rescue; MF, one-month post-rescue.

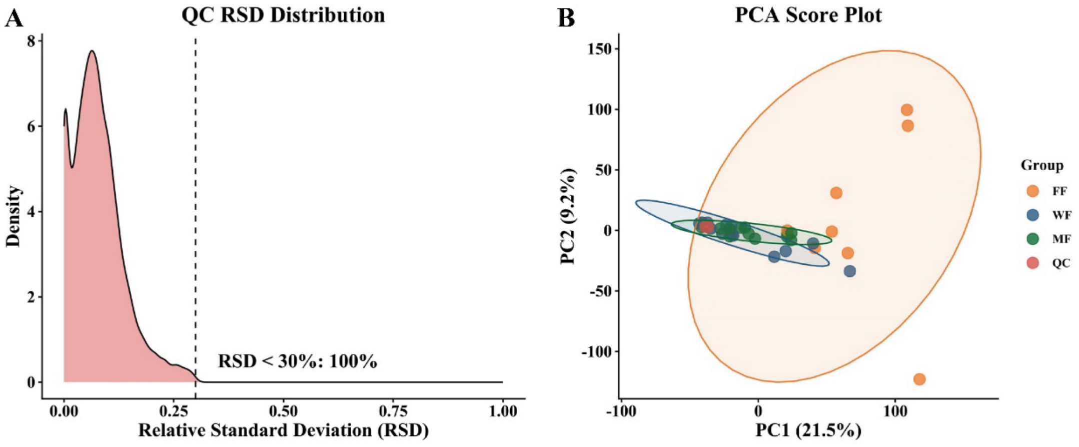

Figure S2. Quality control assessment of untargeted metabolomics data. (A) Distribution of Relative Standard Deviation (RSD) for metabolic features in QC samples, showing 100% of features with RSD < 30%. (B) Unsupervised PCA score plot of all samples. Tight clustering of QC samples (red) indicates high instrumental stability and data reproducibility. FF, first abnormal fecal stage; WF, one-week post-rescue; MF, one-month post-rescue.

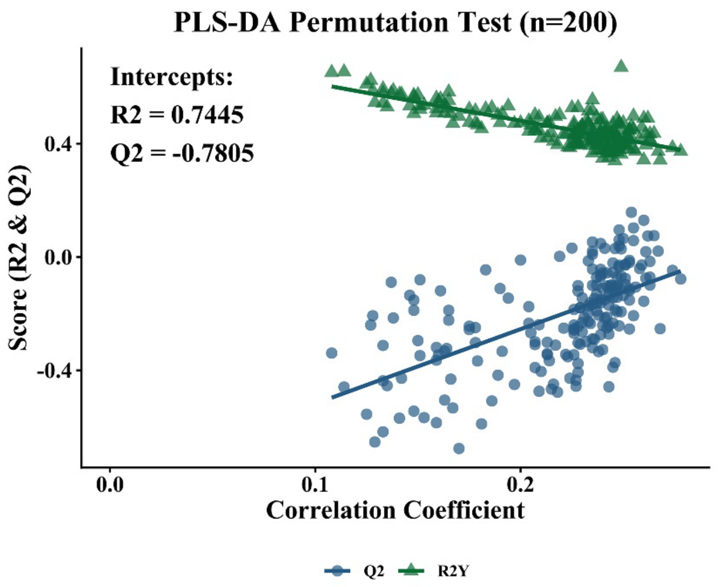

Figure S3. Permutation test of the PLS-DA model (n = 200). The plot shows the correlation between the original and permuted class labels (x-axis) versus the R2 and Q2 values (y-axis).

Supplemental Tables

Table S1. Fecal sample information for rescued Sunda pangolins

Individual ID	Sex	Developmental stage	FF stage sample IDs	WF stage sample IDs	MF stage sample IDs	Total
M1	Male	Adult	FF1, FF3	WF1	MF1, MF2	5
M2	Male	Adult	FF2, FF4	WF2, WF3	MF3, MF4	6
M3	Male	Adult	FF5	WF4, WF5	MF5, MF6	5
M4	Male	Adult	FF6, FF7	WF6, WF7	MF7, MF8	6
M5	Female	Adult	FF8	WF8, WF9	MF9, MF10	5
M6	Female	Adult	FF9	WF10–WF13	MF11, MF12	7

Notes: FF, first abnormal fecal stage; WF, one-week post-rescue stage; MF, one-month post-rescue stage.

Table S2. Detailed sequencing statistics of 16S rRNA gene amplicons for all samples

Sample ID	Raw Reads	Clean Reads	Clean Data (MB)	Effective Rate (%)
FF1	154,614	154,072	42.86	99.86
FF2	154,780	154,360	42.86	99.87
FF3	155,136	154,694	42.86	99.84
FF4	155,674	155,162	42.86	99.87
FF5	155,132	154,614	42.86	99.88
FF6	155,338	154,844	42.86	99.89
FF7	155,586	155,140	42.86	99.89
FF8	155,370	154,866	42.86	99.89
FF9	155,050	154,548	42.86	99.91
WF1	155,554	154,892	42.86	99.87
WF2	156,270	155,734	42.86	99.87
WF3	154,032	153,502	42.86	99.88
WF4	156,656	156,052	42.86	99.83
WF5	155,838	155,150	42.86	99.87
WF6	154,550	154,090	42.86	99.85
WF7	154,882	154,336	42.86	99.86
WF8	156,128	155,676	42.86	99.86
WF9	154,018	153,252	42.86	99.87
WF10	155,430	154,914	42.86	99.86
WF11	155,632	155,186	42.86	99.86
WF12	154,764	154,056	42.86	99.88
WF13	155,008	154,380	42.86	99.83
MF1	154,188	153,508	42.86	99.88
MF2	155,352	154,954	42.86	99.83
MF3	154,294	153,786	42.86	99.87
MF4	155,696	155,202	42.86	99.85
MF5	155,052	154,312	42.86	99.89
MF6	156,436	155,788	42.86	99.84
MF7	154,846	154,282	42.86	99.91
MF8	156,610	156,034	42.86	99.86
MF9	155,920	155,478	42.86	99.86
MF10	155,928	155,424	42.86	99.89
MF11	156,776	156,304	42.86	99.87
MF12	155,110	154,660	42.86	99.83

Table S3. Summary of metagenomic sequencing data quality statistics for all samples

Sample ID	Raw Data (GB)	Valid Data (GB)	Q30 (%)	GC Content (%)	Host Rate (%)
FF1	15.60	8.69	93.20	38.80	13.85
FF2	11.09	8.94	94.28	48.90	11.02
FF3	11.18	8.62	94.43	45.38	14.15
FF4	10.61	9.51	94.76	40.43	5.52
FF5	10.43	10.02	93.77	48.13	0.06
FF6	11.39	10.03	94.81	46.09	0.09
FF7	11.20	9.64	94.10	49.97	4.09
FF8	10.67	10.01	93.88	44.31	0.36
FF9	10.64	7.47	94.89	44.12	25.64
WF1	14.73	9.02	93.28	37.32	9.97
WF2	10.64	9.97	94.16	48.40	0.72
WF3	12.25	9.90	95.19	40.53	1.21
WF4	10.62	8.89	94.80	39.06	11.40
WF5	11.15	10.05	94.92	44.71	0.05
WF6	10.98	10.05	95.06	45.91	0.05
WF7	10.68	8.43	94.95	43.67	16.09
WF8	11.35	10.01	94.54	49.12	0.20
WF9	10.57	9.91	94.70	41.45	1.35
WF10	10.63	8.23	94.50	48.09	17.97
WF11	11.17	9.67	94.89	44.53	3.70
WF12	10.73	10.05	94.92	43.34	0.07
WF13	11.82	7.00	95.03	40.69	30.22
MF1	10.90	10.07	94.88	44.99	0.07
MF2	16.48	10.01	95.01	51.43	0.05
MF3	18.36	10.03	93.76	40.47	0.22
MF4	16.60	10.01	93.34	38.66	0.68
MF5	10.75	10.02	94.55	48.92	0.13
MF6	11.48	9.96	95.15	43.83	0.68
MF7	11.11	10.02	94.87	40.50	0.10
MF8	10.72	10.06	94.94	49.83	0.02
MF9	11.47	10.03	95.19	42.85	0.05
MF10	11.14	10.06	94.65	48.73	0.02
MF11	11.50	9.20	94.72	47.83	8.27
MF12	11.66	10.04	94.83	42.84	0.06
